# Survival in Metastatic Renal Cell Carcinoma Patients Treated With Sunitinib With or Without Cryoablation

**DOI:** 10.3389/fonc.2021.762547

**Published:** 2021-10-20

**Authors:** Cheng-Yuan Gu, Jun-jie Wang, Hai-Liang Zhang, Guo-Hai Shi, Ding-Wei Ye

**Affiliations:** ^1^ Department of Urology, Fudan University Shanghai Cancer Center, Shanghai, China; ^2^ Department of Oncology, Shanghai Medical College, Fudan University, Shanghai, China

**Keywords:** metastatic renal cell cancer carcinoma, cryoablation, targeted therapy, local treatment, sunitinib

## Abstract

**Background:**

Percutaneous cryoablation (PCA) has emerged as an alternative to extirpative management of small renal masses in select patients. In recent years, the use of targeted therapies has become mainstream, while the role of PCA in treating primary tumor is not well established among patients with metastatic renal cell carcinoma (mRCC). We sought to evaluate how mRCC patients react to PCA in combination with sunitinib.

**Methods:**

We retrospectively identified patients with mRCC (primary tumor diameter ≤ 7 cm) treated with sunitinib between 2013 and 2019. These patients were categorized by initial treatment (cryoablation followed by sunitinib versus sunitinib only). Oncological outcomes and rate of adverse events were compared.

**Results:**

Of the 178 patients analyzed, 65 underwent PCA prior to sunitinib. The median overall survival (OS) in the PCA-sunitinib group was 31.7 months (95% CI; 26.1-37.3), better than the sunitinib-only group, which reported a median OS of 19.8 months (95% CI; 17.1-22.4) (*p* < 0.001). The median progression-free survival (PFS) in patients treated with PCA-sunitinib versus sunitinib alone was 13.8 months (95% CI; 10.0-17.6) versus 7.2 months (95% CI: 6.1-8.3) (*p* < 0.005). No significant differences in adverse events were observed (*p* > 0.05).

**Conclusions:**

PCA combined with sunitinib is associated with better survival outcomes than sunitinib alone in patients with mRCC. Careful patient selection remains warranted. These results should inform future prospective trials.

## Background

As a treatment option for cT1 renal cell carcinoma (RCC), percutaneous cryoablation (PCA) has become increasingly common. Ablation was included in the treatment guidelines for early stage RCC published by the American Urologic Association (AUA) in 2009 (1). AUA guidelines and the European Association of Urology guidelines recommended thermal ablation for patients with stage 1 tumors who are not suitable for surgery (2). The National Comprehensive Cancer Network also listed ablation as a recommended treatment in its 2018 guidelines (3). These recommendations were made in light of the similar oncologic outcomes for cT1 renal masses treated by thermal ablation and PN. Moreover, the approach of TA influences the oncologic outcomes (4–7).

Targeted therapy combined with cytoreductive nephrectomy have established roles in the first-line management of mRCC. Many retrospective studies have demonstrated the relative benefits of nephrectomy in patients who received targeted therapy (8, 9). In a systematic meta-analysis that aimed to assess the efficacy of nephrectomy in patients who received targeted therapy, nephrectomy combined with targeted therapy was shown to be associated with superior overall survival benefit than targeted therapy only (10). A U.S. health database analysis revealed an association between initial nephrectomy and improved survival compared to initial targeted therapy (11).

As an alternate approach for tumors < 7 cm, it remains unclear whether PCA before sunitinib provides any benefits to mRCC patients who have no complaints related to their primary tumor. To date, only one study investigated and reported that cryoablation combined with sorafenib had better clinical efficacy against advanced RCC to sorafenib-only (12).

Since little data was available on this topic, we sought to evaluate the benefits of PCA followed by targeted therapy in patients with mRCC relative to targeted therapy alone.

## Methods

### Patients

This retrospective study had been approved by the institutional review board of Fudan University Shanghai Cancer Center. Informed consent was obtained from all patients. All patients with primary mRCC at diagnosis, who were treated with PCA combined with sunitinib or sunitinib only for primary renal mass at our institution between January 2013 and December 2017 were identified. The PCA-sunitinib group included patients who received both sunitinib and PCA, regardless of the order of treatment. The sunitinib group included patients who only received sunitinib. Exclusion criteria were death from non-tumor causes and combination with other therapies during treatment.

Patients with imaging results that suggested RCC were evaluated and referred by urologists or oncologists and presented to the interventional radiology ablation clinic. Indications for PCA included patient comorbidity and surgical risk, history of nephrectomy or otherwise impaired baseline renal function, multiple tumors, tumor size and location, and patient preference. Cryoablation was mostly used to treat stage I RCC (ideally smaller than 7 cm in diameter) in patients who were considered ineligible for surgical resection.

### Treatment

All ablations were performed by operators guided by CT scan with local anesthesia. The number of cryoneedles was determined by tumor diameter. A biopsy was necessary immediately before ablation if the patient had not undergone a diagnostic biopsy before. The procedure aimed to create an ice ball exceeding the tumor margin by at least 5 mm. A baseline noncontrast CT scan was performed with the patient lying prone. The cryoablation protocol started with a 10-minute freeze, then a 10-minute thaw, and ended with a 10-minute refreeze. Manufacturers of the needles used in the cryoablation procedures include Galil Medical (Yokneam, Israel) and Endocare (Irvine, Calif). At the end of the procedure, the needles were removed. This was followed by a noncontrast CT to detect any immediate complications.

Sunitinib therapy started 1 week after cryoablation and lasted until tumor progression or patient death. Both patient groups were initially given sunitinib at 50 mg per day in 6-week cycles of 4 weeks on followed by 2 weeks off. In case of adverse events, dose reduction or discontinuation was allowed.

### Follow-Up

Electronic medical records and radiology results as of December 2017 were reviewed. Patients were followed up in clinics with lab and imaging examination capabilities. CT or MRI scans were repeated at 1, 3, 6, and 12 months after PCA and every 12 months thereafter. CT scans were typically performed with contrast enhancement and image reconstruction in the coronal or sagittal planes, while MRI scans were performed without contrast enhancement to accommodate patients who had poor renal function or allergy to iodine.

### Statistical Analysis

The features by treatment group was compared using the Wilcoxon rank sum, Kruskal Wallis, Fisher exact and chi-square tests. The Kaplan-Meier method was used to estimate overall survival, disease-specific survival, and recurrence-free survival. The followed-up period for recurrence-free survival started from the date of treatment and ended on the date of recurrence, last follow-up for patients treated with PCA, or last imaging for patients treated with ablation. The impact of treatment on patients were assessed using the Cox proportional hazards regression models and reported as hazard ratios (HRs) with 95% confidence intervals (CIs). All statistical analyses were carried out with the SPSS software, version 26.0 (IBM Corp, Armonk, NY, USA).

## Results

The study included 65 patients who were treated with PCA in combination with sunitinib and 113 patients who were given sunitinib only. Key baseline characteristics of the patients and tumors involved are shown in **Table 1**. With the exception of Charlson comorbidity index, no significant differences in all other variables were observed between the two groups.

**Table 1:** Baseline characteristics (SPSS21.0).

Characteristics	PCA- sunitinib (65)	Sunitinib-alone (113)	*P*
**Sex-no. (%)**			0.862
Male	48 (73.8)	81 (71.7)	
Female	17 (26.2)	32 (28.3)	
**Median age(range)-yr**	63 (61-68)	60 (58-62)	0.092
**Histology-no. (%)**			0.294
Clear cell	51 (78.5)	80 (70.8)	
Others	14 (21.5)	33 (29.2)	
**MSKCC risk category-no. (%)**			0.523
Low Risk	2 (3.0)	1 (0.9)	
Intermediate Risk	43 (66.2)	79 (69.9)	
Poor Risk	20 (30.8)	33 (29.2)	
**IMDC risk category-no. (%)**			0.171
Low Risk	2 (3.1)	0 (0)	
Intermediate Risk	34 (52.3)	60 (53.1)	
Poor Risk	29 (44.6)	53 (46.9)	
**ECOG performance-status score-no. (%)**			0.35
0	28 (43.1)	58 (51.3)	
1	37 (56.9)	55 (48.7)	
**Fuhrman grade of renal-cell carcinoma-no. (%)**			0.641
1 or 2	30 (46.2)	57 (50.4)	
3 or 4	35 (53.8)	56 (49.6)	
**Tumor stage-no. (%)**			0.119
T1a T1b	42 (64.6) 23 (35.4)	59 (52.2) 54 (47.8)	
**Median tumor size(range)-cm**	3.5 (3.2-3.8)	3.8 (3.5-4.8)	0.057
**Node stage-no. (%)**			0.622
N0	23 (35.4)	45 (39.8)	
N1	26 (40.0)	37 (32.7)	
TX	16 (24.6)	31 (27.4)	
**Metastatic sites-no. (%)**			0.293
1	23 (35.4)	51 (45.1)	
2	23 (35.4)	42 (37.2)	
3	14 (21.5)	16 (14.2)	
4	5 (7.7)	4 (3.5)	
**Location of metastases-no. (%)**			
Lung	45 (69.2)	65 (57.5)	0.150
Bone	30 (46.2)	38 (33.6)	0.111
Lymph nodes	26 (40.0)	32 (28.3)	0.135
Others	35 (53.8)	53 (46.9)	0.437

**Figure 1:**
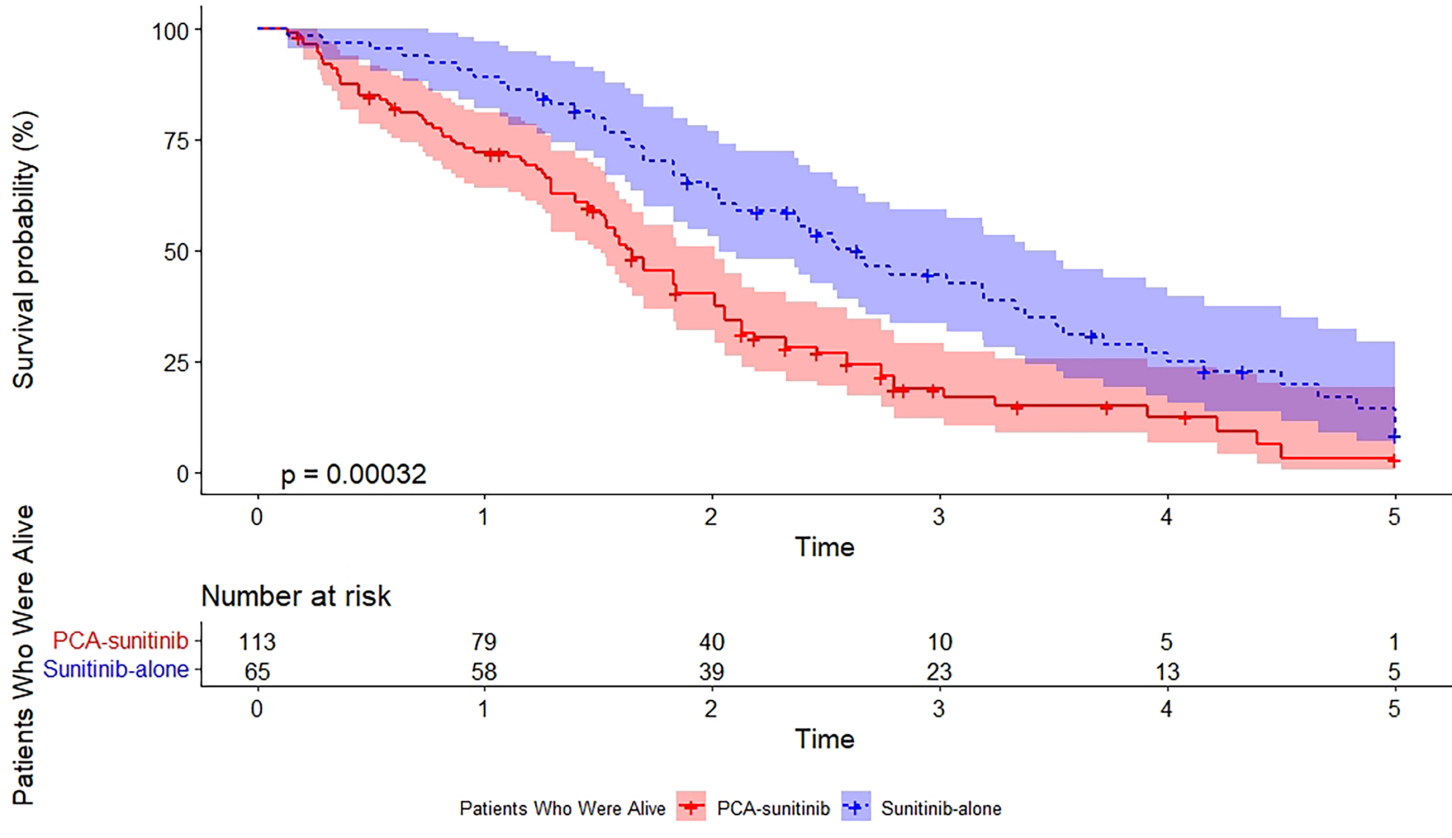
Kaplan-Meier estimate of overall survival.

**Figure 2:**
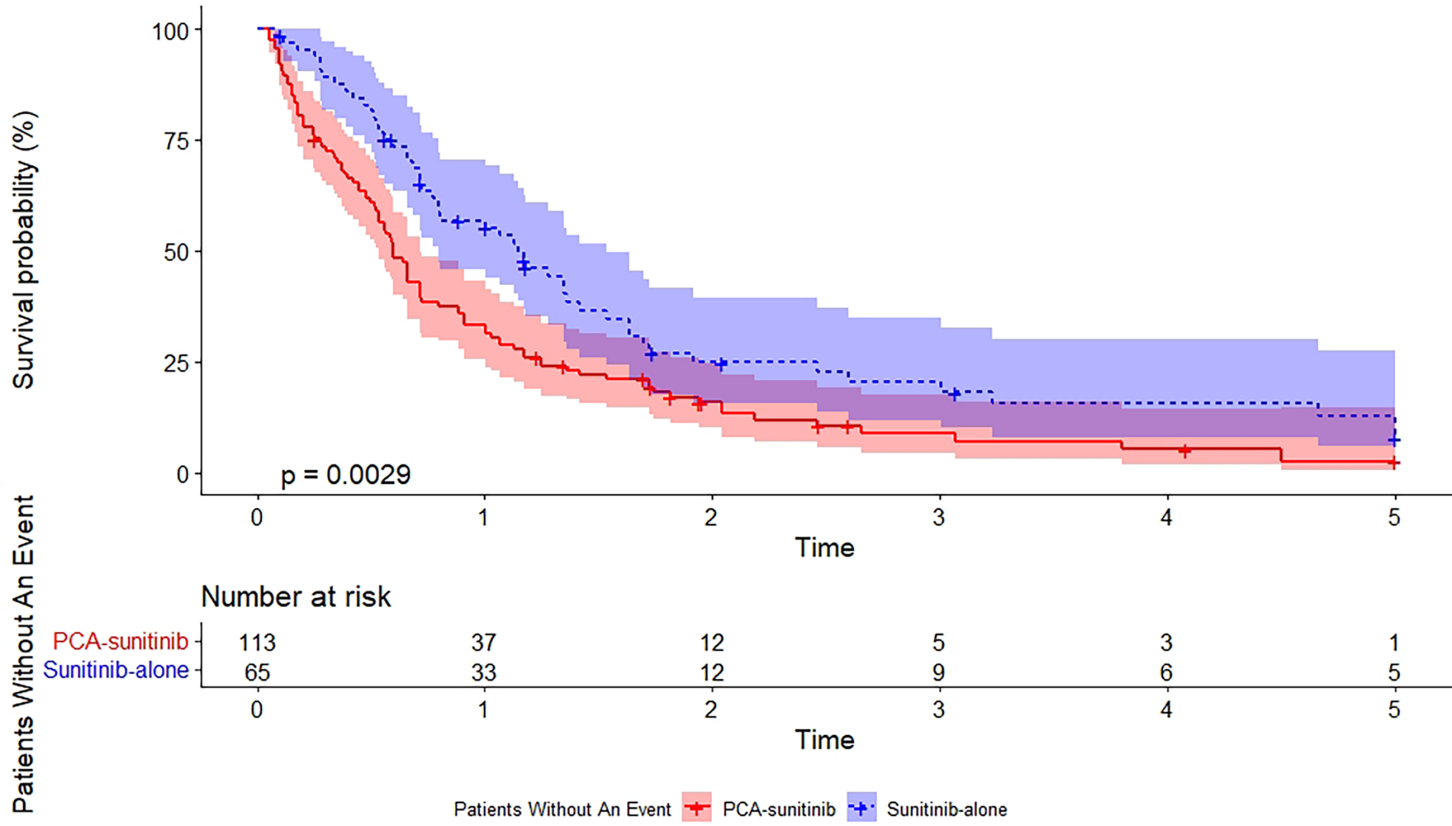
Kaplan-Meier estimate of progression-free survival.

As of the data cutoff date (December 12, 2017), the median follow-up period was 50.0 months [95% confidence interval (CI), 40.7 to 59.3; range, 1.6 to 60.0]. During this period, 39 deaths had occurred, including 15 in the PCA-sunitinib group and 24 in the sunitinib-only group. Median OS in the PCA-sunitinib group was 31.7 months (95% CI; 26.1-37.3), compared with 19.8 months (95% CI; 17.1-22.4) in the sunitinib-only group (**Figure 1**). Median progression-free survival (PFS) in the PCA-sunitinib group was 13.8 months (95% CI: 10.0-17.6), compared with 7.2 months (95% CI: 6.1-8.3) in the sunitinib-only group (**Figure 2**). Cox proportional hazards analysis revealed PCA-sunitinib was associated with improved PFS (HR: 0.612, 95% CI: 0.435-0.862, p < 0.005) and OS (HR: 0.525, 95% CI: 0.367-0.752, p < 0.001).

The objective response rate was significantly higher in the PCA-sunitinib group (58.46% versus 41.59% in the sunitinib-only group, *p* = 0.042), and the disease control rate also showed improvement in the PCA -sunitinib group than in the sunitinib-only group (92.31% vs 80.53%, *p* = 0.049) (**Table 2**). There were 38/65 patients who achieved partial response in PCA-sunitinib group while 47/113 patients in sunitinib alone group. There were 22/65 patients who had stable disease in PCA-sunitinib group while 44/113 patients in sunitinib alone group. There were 5/65 patients who suffered progression of disease in PCA-sunitinib group while 22/113 patients in sunitinib alone group.

**Table 2:** Tumor response outcomes.

Response	PCA-sunitinib (65)	Sunitinib-alone (113)	*P*
**Best overall response-no./total**			0.038
Partial response	38/65	47/113	
Stable disease	22/65	44/113	
Progression of disease	5/65	22/113	
**Objective response rate-%**	58.46%	41.59%	
**Disease control rate-%**	92.31%	80.53%	

Overall, 14.0% of the cases experienced grade 3 or 4 adverse events; 9 patients (13.8%) in the PCA-sunitinib group and 16 (14.2%) in the sunitinib-only group reported grade 3 or 4 adverse events (**Table 3**). Among the 178 patients, the most common adverse events that were observed included hand–foot syndrome (in 101 patients), diarrhea (in 58), thrombocytopenia (in 50), and neutropenia (in 45). There were not evident differences in toxic effects between the two groups.

**Table 3:** Summary of severe adverse events in sunitinib-treated patients.

Event-no. (%)	PCA-sunitinib (65)	Sunitinib-alone (113)	*P*
Any adverse event of grade 3 or 4	9 (13.8)	16 (14.2)	1.000
Hand–foot syndrome	35 (53.8)	66 (58.4)	0.395
Rash	17 (26.2)	26 (23.0)	0.894
Hair loss	11 (16.9)	23 (20.4)	0.677
Diarrhea	19 (29.2)	39 (34.5)	0.523
Severe high blood pressure	11 (16.9)	20 (17.7)	0.844
Inflammation of mucosa	14 (21.5)	17 (15.0)	0.546
Fever or allergy	2 (3.1)	5 (4.4)	1.000
Liver damage	14 (21.5)	15 (13.3)	0.815
Asthenia	14 (21.5)	25 (22.1)	1.000
Hypothyroidism	7 (10.8)	14 (12.4)	0.814
Neutropenia	19 (29.2)	26 (23.0)	0.375
Thrombocytopenia	18 (27.7)	32 (28.3)	1.000

## Discussion

Evidence for the role of PCA in treating primary renal tumor in patients with metastatic disease is limited, although local therapy has been proven to prolong OS in other primary metastasized malignancies (13–17). We compared the oncologic outcomes of PCA plus sunitinib or sunitinib only in mRCC patients. Our results demonstrated a significant OS advantage in patients receiving PCA combined with sunitinib over sunitinib only. Similar to our study, Liu et al. reported that cryoablation-sorafenib led to improved OS and PFS compared with sorafenib-only therapy (OS: 36 months vs.29 months, *p* < 0.001; PFS: 20 months vs. 12 months, *p* < 0.005) (12). Our OS and PFS is within the range of results reported for cytoreductive nephrectomy (9, 18–21). High quality, comparative data on the cancer control outcomes of cytoreductive nephrectomy-sunitinib vs sunitinib alone can be obtained from the phase 3 randomized SURTIME trial (18). The median OS was 18.4 months in the sunitinib-only group, versus 13.9 months in the nephrectomy–sunitinib group. The median PFS was longer in the sunitinib-only group (8.3 months, 95% CI: 6.2-9.9) than in the nephrectomy–sunitinib group (7.2 months, 95% CI: 6.7-8.5). A National Cancer Database analysis which involved 4223 mRCC patients receiving nephrectomy in combination with targeted therapy showed that cytoreductive nephrectomy may provide OS benefits to patients treated with targeted therapy (17.1 months vs. 7.7 months, *p* < 0.001) (8). Of note, most of the patients in the current study had tumors of 4 cm or less in size. These data, together with a growing body of evidence, has showed that surgical and ablative treatments offer comparable overall and cancer-specific survival outcomes for mRCC patients.

There are reasonable arguments in favor of combining local therapy and systemic treatment for mRCC patients due to its ability to improve tumor control and alleviate disease progression. In a number of malignancies, decreasing tumor burden through cytoreductive cryoablation can have positive effect on survival. In particular, studies have demonstrated that cryoablation leads to better survival in patients with metastatic hepatocellular carcinoma (22), lung cancer (23), and melanoma (24), on top of strengthening tumor response to systemic therapy. Several studies have reported synergies between cryoablation and immune response. The tumor tissue remains in place after ablation, releasing factors that stimulate the immune system. Cryoablation induces a short-lived immune response and upregulates the expression of PD-1 and PD-L1 within the distant tumor microenvironment in RCC (25). Necrotic cells produced by cryoablation send out danger signals that can activate immune response. As a result, both immunostimulatory and immunosuppressive responses may be triggered by cryoablation. Following cryoablation of the RCC tissue, an infiltration of neutrophils, CD4+ and CD8+ T cells and macrophages was observed (26). Another study on the efficacy of cryoablation relative to surgery showed decreased tumor growth rates, as the tumor cells were re-challenged by significantly increased T cells in the peripheral blood stream after cryoablation (27). These findings point to the potential of cryoablation to enhance tumor immunity to a greater extent than targeted therapy alone.

There were not significant differences in the severity and incidence of adverse events were observed between the PCA-sunitinib and sunitinib-alone groups (*p* > 0.05). Mershon JP et al. summarized the oncologic outcomes, complication rates, procedural differences and renal function of cryoablation, radiofrequency ablation, and microwave ablation as management strategy for small renal masses (28). Similar with other ablative techniques, cryoablation is a safe and effective management option for small renal masses in select patients, particularly in those with multiple tumors and/or those unable or unwilling to undergo more invasive surgery. The main advantages of PCA include its minimally invasive nature and ability to preserve renal function as much as possible. Guided by CT or MRI, the growing ice ball is directly visible, including its precise margins, which helps provide a high level of treatment efficacy and safety. Although surgical resection is currently the standard treatment for mRCC patients, many of them are not suitable surgical candidates due to their age and comorbidity, which entail high surgical risks. For these patients with low performance status scores, cryoablation is a more appropriate option.

The current study has some limitations. First, the retrospective nature of the analyses inevitably leads to a selection bias commonly found in non-prospective, non-randomized studies. Although most patient and tumor characteristics were matched in two groups, selection bias or confounders inherent in retrospective studies cannot be ruled out, which we did not control for. Furthermore, though this study it is limited by a small sample size with relatively short follow-up, though its size is comparable to that of other studies with a specific focus on RCC cryoablation. This should be taken into consideration in the interpretation of our results. In future studies, data can be collected from multiple institutions in a prospective manner, in order to have a more robust analysis of oncologic outcomes.

## Conclusion

In conclusion, cryoablation has demonstrated efficacy in the local control of various cancer types. The discovery of synergies between local ablative techniques and systemic treatments has opened up exciting new opportunities in the field of interventional oncology. In particular, cryoablation can release antigens recognized by the immune system, hence stimulating specific immune response against the tumor cells. In the current study, PCA-sunitinib therapy is found to be associated with longer PFS and OS in mRCC patients versus sunitinib alone. Careful patient selection is still key in assessing whether or not patients will benefit from cryoablation.

## Data Availability Statement

The raw data supporting the conclusions of this article will be made available by the authors, without undue reservation.

## Ethics Statement

The studies involving human participants were reviewed and approved by Fudan University Shanghai Cancer Center. The patients/participants provided their written informed consent to participate in this study.

## Author Contributions

C-YG and J-jW analyzed and interpreted the data, and was the major contributors in writing the manuscript. H-LZ performed the ablations process. G-HS and D-WY conceptualized the study design, validated the results, performed project administration. All authors contributed to the article and approved the submitted version.

## Funding

This study was supported by Shanghai Natural Science Foundation (No. 20ZR1412500) and Fudan University Shanghai Cancer Center Foundation (No. YJMS202004).

## Conflict of Interest

The authors declare that the research was conducted in the absence of any commercial or financial relationships that could be construed as a potential conflict of interest.

## Publisher’s Note

All claims expressed in this article are solely those of the authors and do not necessarily represent those of their affiliated organizations, or those of the publisher, the editors and the reviewers. Any product that may be evaluated in this article, or claim that may be made by its manufacturer, is not guaranteed or endorsed by the publisher.

## Abbreviations

mRCC, metastatic renal cell carcinoma; PCA, Percutaneous cryoablation; PFS, progression-free survival; OS, overall survival.
